# The Insulin-like Growth Factor Axis, Adipokines, Physical Activity, and Obesity in Relation to Breast Cancer Incidence and Recurrence

**DOI:** 10.5539/cco.v4n2p24

**Published:** 2015-07-20

**Authors:** Steven S. Coughlin, Selina A. Smith

**Affiliations:** 1 Department of Epidemiology, Rollins School of Public Health, Emory University, Atlanta, GA, USA; 2 Institute of Public and Preventive Health, and Department of Family Medicine, Medical College of Georgia, Georgia Regents University, Augusta, GA, USA

**Keywords:** adiponectin, African Americans, breast cancer, insulin-like growth factor, leptin, obesity, physical activity, survival

## Abstract

**Background:**

Obesity, a risk factor for the development of postmenopausal breast cancer and certain other cancer types, has also been associated with poorer response to cancer therapy and cancer recurrence. The insulin-like growth factor (IGF) axis also influences cancer risk.

**Methods:**

In this commentary, we consider the literature on IGF and its binding proteins and the risk of breast cancer, along with effects of obesity, adipokines, and insulin resistance on breast cancer, and the potential for lifestyle interventions to address weight gain and physical inactivity among at-risk women.

**Results:**

Greater body fatness is associated with a higher risk of postmenopausal breast cancer. The association may be explained, in part, by hyperinsulinemia and alterations in adipokines and estrogens. Nutrition, energy balance, and levels of physical activity are determinants of IGF bioactivity. Alterations in the IGF axis can increase cancer risk and progression. Results from epidemiologic studies indicate that higher circulating levels of IGF-I are associated with an increased risk of breast cancer.

**Conclusions:**

Intervention studies are needed to determine how to sustain the positive effects of exercise over time and to identify the optimal mode, intensity, frequency, duration, and timing of exercise for breast cancer survivors, including important subgroups of survivors such as African American and Hispanic women. Future epidemiologic studies of the relationships between the IGF axis and breast cancer should include adequate numbers of African American women, Hispanic women, and other minority women who have been underrepresented in studies completed to date.

## 1. Introduction

Obesity, a risk factor for the development of postmenopausal breast cancer and certain other cancer types, has also been associated with poorer response to cancer therapy and cancer recurrence (Surmacz & Otvos, 2015). The increasing prevalence of obesity in the U.S. population, the independent association of obesity with cancer incidence, and the potential involvement of obesity in the recurrence of cancer have prompted interest in understanding the biological mechanisms that underlie obesity-cancer linkages (Vansaun, 2013). Obesity and physical inactivity are determinants of hyperinsulinema and insulin resistance (Giovannucci & Michaud, 2007), and obesity influences the amount of free insulin-like growth factor (IGF-I) available to cells (Coughlin & Giovanucci, 2012). Hyerinsulinemia may be an underlying factor in chronic conditions such as type 2 diabetes mellitus, obesity, and certain forms of cancer, including breast cancer.

Weight gain and physical inactivity can occur following breast cancer treatment. For breast cancer survivors, cancer-related fatigue is a common symptom (Meneses-Echavez, Gonzalez-Jimenez, & Ramirez-Velez, 2015). Among women who have already been diagnosed with breast cancer, obesity is associated with breast cancer recurrence and poorer survival (Protani, Coory, & Martin, 2010). Maintaining a healthy body weight reduces a woman’s risk of cancer recurrence, diabetes and cardiovascular diseases (Thompson et al., 2012).

In the U.S., breast cancer survival rates are lower among African American women than white women (Coughlin & Cypel, 2013). Modifiable lifestyle factors related to energy balance and physical activity may contribute to this disparity. African American breast cancer survivors are less likely to report adherence to American Cancer Society recommendations for physical activity and nutrition (Kushi et al., 2012).

In this commentary, we consider the literature on IGF, a mitogen in the mammary gland, and its binding proteins and risk of breast cancer. We also examine the roles played by obesity, adipokines secreted by adipose tissue, and insulin resistance on breast cancer carcinogenesis and the potential for lifestyle interventions to address weight gain and physical inactivity among at-risk women and breast cancer survivors, including African American women. We consider the interplay been these factors in influencing risk of breast cancer incidence and recurrence. Finally, we discuss remaining challenges in this area and offer suggestions for additional research.

## 2. Insulin-Like Growth Factor and Its Binding Proteins and Risk of Breast Cancer

Development and differentiation of the normal mammary gland are dependent on the expression of locally produced growth factors (e.g., IGF-I) and systemically acting hormones. Two important factors involved in cellular and tissue function are the estrogen and insulin-like growth factor axes (Hawsawi et al., 2013). The interaction of these pathways at transcriptional and translational levels regulates the expression and activity of genes involved in the development of the mammary gland. Estradiol, acting through estrogen receptors, is essential for the normal function of the mammary gland but can also promote the growth of breast cancer (Hawsawi et al., 2013). Hence, regimens for the treatment of breast cancer commonly include selective estrogen receptor modulators, aromatase inhibitors, or selective estrogen receptor down-regulators. Breast tumors can develop resistance to anti-estrogen therapies, however, and other pathways (e.g., the IGF axis) are likely to be involved in breast cancer development and response to treatment. With respect to breast tumor development, experimental studies suggest that there appears to be a synergistic effect of estrogen receptor activation and increased IGF-I signaling (Kaaks et al., 2014).

Obesity influences the amount of free IGF-I available to cells, but is not a determinant of total IGF-I in the blood (Coughlin & Giovanucci, 2012). Hyerinsulinemia is thought to be an underlying factor in breast cancer incidence. Higher insulin levels may contribute to increased tumor growth (Coughlin & Giovanucci, 2012). Increases in circulating levels of IGF-I) have been observed in some epidemiologic studies of premenopausal breast cancer. IGF-I is an important mitogen in the mammary gland (Christopoulos, Msaouel & Koutsilieris, 2015). When IGF-I binds to its cognate receptor (IGF-1R), it triggers a signaling cascade that leads to proliferative and anti-apoptotic events. The IGF-I system is involved in breast cancer development, progression, and metastasis (Christopoulos, Msaouel, & Koutsilieris, 2015).

The relationship between pre-diagnostic levels of IGF-I and IGF binding protein-3 (IGFBP-3) and breast cancer risk was examined in a meta-analysis of data from 17 prospective studies conducted in 12 countries (Endogenous Hormones and Breast Cancer Collaborative Group, 2010). The overall odds ratio [OR] for breast cancer incidence was 1.28 (95% confidence interval [CI] 1.14-1.44). The positive association with IGF-I, which was not substantially modified by IGFBP-3 or by menopausal status, was limited to estrogen-receptor-positive breast cancers. Results from epidemiologic studies indicate that there are potentially modifiable determinants of circulating IGF-I concentrations including intake of dietary protein (Bradbury et al. 2015). Kaaks et al. (2014) conducted a case-control study of IGF-I and breast cancer incidence by age and hormone receptor status, nested within the prospective European EPIC cohort (938 breast cancer cases and 1,394 matched controls). IGF-I levels were positively associated with the risk of estrogen receptor-positive tumors overall (pre- and postmenopausal women combined, adjusted OR = 1.41, 95% CI 1.01-1.98 for the highest vs. lowest quartile) and among women who were diagnosed with breast cancer at 50 years or older (adjusted OR = 1.38, 95% CI 1.01-1.89), but not with receptor-positive disease diagnosed at an earlier age. No statistically significant associations were observed for estrogen receptor negative tumors overall or by age at diagnosis (Kaaks et al., 2014).

Although some studies have suggested that IGF-I is positively associated with risk of breast cancer incidence among premenopausal women, the results of epidemiologic studies have been inconsistent. Schernhammer et al. (2006) conducted a nested, case-control study of IGF-I, insulin-like binding protein-1 (IGFBP-1) and IGFBP-3 and breast cancer incidence in the Nurses Health Study II cohort, which mainly consists of premenopausal women. Plasma levels of IGF-I and its binding proteins were measured using prediagnostic samples obtained from 317 women diagnosed with invasive or in situ breast cancer and 634 matched controls. Overall, plasma levels of IGF-I, IGFBP-1 and IGFBP-3 were not associated with breast cancer risk. To examine the relationships between IGF-I and breast cancer incidence among premenopausal women, Rinaldi et al. (2006) conducted a pooled analysis of data from three prospective studies in New York; northern Sweden; and Milan, Italy. Statistically significant, positive associations were observed between IGF-I and IGFBP-3 and breast cancer risk among younger women.

Prognostic studies have shown that expression of IGF-1R, the receptor for IGF-I, is predictive of improved survival and that its expression is related to hormone receptor status (Yan, Jiao, Li, Li & Zou, 2005; Shin et al., 2014). However, IGF-1R is a favorable prognostic indicator only in hormone receptor-positive breast cancers. IGF-1R positivity reflects a well-differentiated tumor with low metastatic tendency (Aaltonen et al., 2014). Among women with triple-negative breast cancers, IGF-1R is a predictor of poorer survival (Hernandez et al., 2015). Hernandez et al. (2015) examined the expression of proteins in the IGF axis and the association with breast cancer survival. In Native Hawaiian patients, IGFBP-2 and IGFBP-3 expression were each independently associated with overall and breast cancer mortality. IGF-1R expression was also positively associated with overall mortality in Native Hawaiians (Hernandez et al., 2015). In Japanese or Caucasian patients, however, no association of expression of IGF proteins and survival was observed.

## 3. Obesity, Adipokines, Insulin Resistance and Breast Cancer Carcinogenesis

Potential mechanisms linking obesity to higher cancer risk include obesity-related insulin resistance, hyperinsulinemia, hyperglycemia, glucose intolerance, and adipocytokine production (Booth, Magnuson, Fouts, & Foster, 2015). Adipose tissue secretes bioactive factors including estrogens and adipokines such as leptin and adiponectin, which mediate metabolism, angiogenesis, and cellular proliferation (Schmidt, Monk, Robinson, & Mourtzakis, 2015). Visceral fat is an active metabolic and endocrine tissue (Golubovic et al., 2013). Through the release of adipokines, adipose tissue influences several physiological functions, including appetite, satiety, energy expenditure, insulin sensitivity, and glucose and lipid metabolism (Bluher & Mantzoros, 2015). The hypertrophy of adipocytes and accumulation of excessive adipose tissue leads to dysregulation and alteration of peripheral tissue metabolism (Booth, Magnuson, Fouts & Foster, 2015). Leptin secretion from adipose tissue is believed to promote breast cancer directly and independently and also through its effects on estrogen and insulin signaling pathways (Surmacz & Otvos, 2015). Leptin promotes breast cancer progression through the activation of mitogenic, antiapoptotic, and metastatic pathways (Surmacz, 2013). Although leptin activates some carcinogenic pathways, adiponectin appears to have a regulatory role in insulin resistance and to exert antineoplastic activities and interfere with leptin-induced processes (Balsan, Vieira, Oliveira, & Portal, 2015). Excess body fat is associated with increased expression of leptin and downregulation of adiponectin (Surmacz & Otvos, 2015). The deregulation of adipokine signaling and altered adipokine hormone secretion from excess adipose is an area of active research into the mechanism and potential treatment (molecular targeting) of obesity-related cancer (Surmacz, 2013; Drew, 2015). In anti-obesity therapeutic interventions, adipokines have potential to be biomarkers of individual treatment success and disease progression (Bluher & Mantzoros, 2015).

Increased body weight and accumulation of central adiposity lead to changes in leptin and adiponectin levels and reduction of insulin sensitivity; weight loss improves insulin sensitivity and decreases risk for many potential complications of obesity (Golubovic et al., 2013). In a study of 90 obese women, a calorie-restricted diet and weight loss of > 5% over a six-month period led to a reduction in leptin levels and an increase in adiponectin levels (p < 0.001). Fasting glucose and insulin levels were also reduced in the research participants (Golubovic et al., 2013). Independent of weight loss, energy expenditure through exercise by improve leptin regulation (Schmidt, Monk, Robinson & Mourtzakis, 2015).

### 1.3 Addressing Weight Gain and Physical Inactivity Among Breast Cancer Survivors

Physical activity and adopting a healthy diet are beneficial both in the general population and among breast cancer survivors (Kushi et al., 2012). Among breast cancer survivors, a variety of approaches, including exercise and weight training, dietary interventions, yoga and mindfulness-based stress reduction, have addressed weight gain, fatigue, and other complaints. Considerable information exists about the effectiveness of such interventions for alleviating distress and improving quality of life among breast cancer survivors (Mishra et al., 2012; Meneses-Echavez, Gonzalez-Jimenez, & Ramirez-Velez, 2015). Exercise (e.g., strength training, resistance training, walking, cycling, yoga, or Tai Chi) improves overall quality of life and cancer-related fatigue (Mishra et al. 2012). A meta-analysis of 9 high-quality studies found that supervised aerobic exercise is more effective than conventional care in improving cancer-related fatigue among breast cancer survivors (standardized mean difference [SMD] = −0.51, 95% confidence interval [CI] −0.81 to −0.21) (Meneses-Echavez, Gonzalez-Jimenez, & Ramirez-Velez, 2015). Similar effects were found for resistance training on cancer related fatigue (SMD = −0.41, 95% CI −0.76 to −0.05).

In a meta-analysis of studies of breast cancer survivors, Lahart et al. (2015) found an inverse relationship between physical activity and all-cause, breast cancer-related death and breast cancer events, supporting the view that physical activity reduces death and breast cancer events among breast cancer survivors. Among older, overweight, and obese cancer survivors, physical activity and improved diet are positively associated with weight loss and higher physical functioning (Morey et al., 2009; Demark-Wahnefried et al., 2012; Kenzik et al., 2015).

In the RENEW trial, which included survivors of breast, colorectal, and prostate cancer aged 65 to 91 years, a tailored intervention consisting of telephone counseling and mailed print materials on diet and exercise delivered over a 12-month period was effective in increasing exercise, improving diet, reducing weight, and increasing quality-of-life (Morey et al., 2009). Lifestyle interventions that focus on physical activity and improved diet reduce functional decline among older cancer survivors (Demark-Wahnefried et al., 2012). Among the many randomized trials of physical activity and/or dietary interventions for breast cancer survivors, however, few have examined the maintenance of outcomes post-intervention, examined mediators of intervention effects, or focused on African American or Hispanic survivors (Spark, Reeves, Fieldsoe, & Eakin, 2013; Short, James, Stacey, & Plotnikoff, 2013).

African American breast cancer survivors are more likely to be obese and less likely to engage in physical activity than white survivors (Spector et al., 2014). In a study of 724 African American and 116 white breast cancer survivors, the African American survivors were more than twice as likely to have diminished physical functioning (odds ratio [OR] = 2.29, 95% CI 1.57, 3.34) (Spector et al., 2014). However, much of this disparity was due to racial differences in body mass index and other variables. A cross-sectional analysis of data from the 2009 Behavioral Risk Factor Surveillance System showed that African American breast cancer survivors were more likely to report obesity but less likely to report heavy alcohol consumption than their white counterparts (White et al. 2009). In a pilot study of 17 African American breast cancer survivors (stage 0 to IIIA) who were within 2 years of completing primary cancer treatments, in which the participants completed weekly exercise logs and received weekly motivational phone calls, a 16-week training program involving home-based aerobic and resistance physical activity improved cardiopulmonary fitness, strength, and functional movement (Spector et al., 2014). Greenlee et al. (2013) completed a randomized controlled trial following 42 Hispanic and African American breast cancer survivors for 6 months in the commercially available Curves program, resulting in weight loss that was not maintained at 6 months post-intervention. The trial was limited by the small sample size, however. A community-based pilot study of walking (Wilson, Porter, Parker, & Kilpatrick, 2005), with 24 African American breast cancer survivors, resulted in increases in steps walked per day and decreases in body mass index, body weight and waist/hip circumferences, with most changes maintained at a 3-month follow-up. Stolley et al. (2009) used a pre-post design to test a 6-month intervention that included one of two weekly sessions dedicated to exercise; participants experienced significant changes in weight, body mass index, and social support. In a 16-week, home-based motivational exercise program for 13 African American breast cancer survivors, there was an increase in total minutes of physical activity post-intervention with improved physical functioning (Bock, Marcus, Pinto & Forsyth, 2001). In this population, larger-scale studies are needed to identify effective approaches to influence physical activity, nutrition, and achieve improvements in health-related quality-of-life.

## 4. Summary and Conclusions

Epidemiologic studies indicate that greater body fatness is associated with a higher risk of certain cancers, including postmenopausal breast cancer and that the positive association may be partly explained, in part, by hyperinsulinemia and alterations in adipokines and estrogens (Nimptsch & Pichon, 2015). Considerable information exists about the effectiveness of interventions, such as exercise, weight training, dietary interventions, yoga and mindfulness-based stress reduction, for alleviating distress and improving quality of life among breast cancer survivors. Additional studies are needed to determine how to sustain the positive effects of exercise over time and to identify the optimal mode, intensity, frequency, duration, and timing of exercise for breast cancer survivors, including important subgroups such as African American and Hispanic women. Intervention studies on nutrition in these populations are also needed.

Nutrition, energy balance, and physical activity levels are important determinants of IGF-I bioactivity. Alterations in the IGF axis, related to physical activity and nutrition, can increase cancer risk and progression (Zielinska, Bahl, Holly & Perks, 2015). This axis is involved in cellular growth, differentiation, proliferation regulation, and apoptosis. Results from epidemiologic studies indicate that higher circulating levels of IGF-I are associated with increased risk of breast cancer but do not support an association between IGFBP-1 and breast cancer. Although some epidemiologic studies support an association between IGFBP-3 and risk of breast cancer among younger women, results to date have been inconsistent. This inconsistency may be accounted for by differences in study design and lack of standardization across assays. IGFBP-3 regulates the bioavailability of IGF-I and is involved in breast cancer prognosis. Preliminary studies indicate that it may be possible to personalize dietary recommendations for breast cancer survivors based on IGF-I receptor status or other molecular characteristics of their tumor tissue (Emond et al., 2014).

There is increasing knowledge about the determinants of racial and ethnic disparities in breast cancer outcomes. It is likely that, in addition to a variety of social, structural, and environmental factors, biological factors influence how breast cancer is expressed (Coughlin & Cypel, 2013). Alterations in IGF signaling induced by obesity and/or other factors may lead to these disparities (Hernandez et al., 2015). However, there is currently a paucity of health intervention studies that include sizeable numbers of African American or Hispanic breast cancer survivors. Future health intervention trials and epidemiologic studies of the relationships between the insulin-growth factor axis and breast cancer or breast cancer recurrence should include adequate numbers of African American, Hispanic, and non-Hispanic white women so that interactive effects by race and ethnicity can be more fully explored. Additional studies are also needed of American Indian, Alaska Native, Asian, and Pacific Islander women at-risk for breast cancer or for recurrence of the disease.

## References

[R1] Aaltonen KE, Rosendahl AH, Olsson H, Malmström P, Hartman L, Fernö M (2014). Association between insulin-like growth factor-1 receptor (IGF1R) negativity and poor prognosis in a cohort of women with primary breast cancer. BMC Cancer.

[R2] Balsan GA, Vieira JL, Oliveira AM, Portal VL (2015). Relationship between adiponectin, obesity and insulin resistance. Revista da Associação Médica Brasileira.

[R3] Bluher M, Mantzoros CS (2015). From leptin to other adipokins in health and disease: Facts and expectations at the beginning of the 21^st^ century. Metabolism.

[R4] Bock BC, Marcus BH, Pinto BM, Forsyth LH (2001). Maintenance of PA following an individualized motivationally tailored intervention. Annals of Behavioral Medicine.

[R5] Booth A, Magnuson A, Fouts J, Foster M (2015). Adipose tissue, obesity and adipokines: Role in cancer promotion. Hormone Molecular Biology & Clinical Investigation.

[R6] Bradbury KE, Balkwill A, Tipper SJ (2015). The association of plasma IGF-I with dietary, lifestyle, anthropometric, and early life factors in postmenopausal women. Growth Hormone IGF Research.

[R7] Calhoun C, Helzlsouer KJ, Gallicchio L (2015). Racial differences in depressive symptoms and self-rated health among breast cancer survivors on aromatase inhibitor therapy. Journal of Psychosocial Oncology.

[R8] Christopoulos PF, Msaouel P, Koutsilieris M (2015). The role of the insulin-like growth factor-1 system in breast cancer. Molecular Cancer.

[R9] Coughlin SS, Giovanucci EL, Shaw KM, Cummings MH (2012). Diabetes and cancer. Diabetes: Chronic Complications.

[R10] Coughlin SS, Cypel Y, Ahmad A (2013). Epidemiology of breast cancer in women. Breast Cancer Metastasis and Drug Resistance: Challenges and Progress.

[R11] Demark-Wahnefried W, Morey MC, Sloane R, Snyder DC, Miller PE, Hartman TG, Cohen HJ (2012). Reach out to enhance wellness home-based diet-exercise intervention promotes reproducible and sustainable long-term improvements in health behaviors, body weight, and physical functioning in older, overweight/obese cancer survivors. Journal of Clinical Oncology.

[R12] Drew JE (2012). Molecular mechanisms linking adipokines to obesity-related colon cancer: focus on leptin. Proceedings Nutrition Society.

[R13] Emond JA, Pierce JP, Natarajan L, Gapuz LR, Nguyen J, Parker BA, Patterson RE (2014). Risk of breast cancer recurrence associated with carbohydrate intake and tissue expression of IGFI receptor. Cancer Epidemiology Biomarkers & Prevention.

[R14] Endogenous Hormones and Breast Cancer Collaborative Group (2010). Insulin-like growth factor I (IGF-I), IGF binding protein 3 (IGFBP3), and breast cancer risk: pooled results from 17 prospective studies. Lancet Oncology.

[R15] Giovannucci E, Michaud D (2007). The role of obesity and related metabolic disturbances in cancers of the colon, prostate, and pancreas. Gastroenterology.

[R16] Golubovic MV, Dimic D, Antic S, Radenković S, Ðinđić B, Jovanović M (2013). Relationship of adipokine to insulin sensitivity and glycemic regulation in obese women—the effect of body weight reduction by caloric restriction. Vojinosanitetski Pregled.

[R17] Greenlee HA, Crew KD, Mata JM, McKinley PS, Rundle AG, Zhang WF, Hershman DL (2013). A pilot randomized controlled trial of a commercial diet and exercise weight loss program in minority BCS. Obesity.

[R18] Hawsawi Y, El-Gendy R, Twelves C (2013). Insulin-like growth factor—oestradiol crosstalk and mammary gland tumourigenesis. Biochimica et Biophysica Acta.

[R19] Hernandez BY, Wilkens LR, Le Marchand L, Horio D, Chong CD, Lo LWM (2015). Differences in IGF-axis protein expression and survival among multiethnic breast cancer patients. Cancer Medicine.

[R20] Kaaks R, Johnson T, Tikk K, Sookthai D, Tjønneland A, Roswall N, Lukanova A (2014). Insulin-like growth factor I and risk of breast cancer by age and hormone receptor status—A prospective study within the EPIC cohort. International Journal of Cancer.

[R21] Kenzik KM, Morey MC, Cohen HJ, Sloane R, Demark-Wahnefried W (2015). Symptoms, weight loss and physical function in a lifestyle intervention study of older cancer survivors. Cancer Epidemiology Biomarkers & Prevention.

[R22] Kushi LH, Doyle C, McCullough M, Rock CL, Demark-Wahnefried W, Bandera EV, The American Cancer Society 2010 Nutrition and Physical Activity Guidelines Advisory Committee (2012). American Cancer Society guidelines on nutrition and physical activity for cancer prevention. CA: A Cancer Journal for Clinicians.

[R23] Lahart IM, Metsios GS, Nevill AM, Carmichael AR (2015). Physical activity, risk of death and recurrence in breast cancer survivors: a systematic review and meta-analysis of epidemiological studies. Acta Oncologica.

[R24] Meneses-Echavez JF, Gonzalez-Jimenez E, Ramirez-Velez R (2015). Effects of supervised exercise on cancer-related fatigue in breast cancer survivors: a systematic review and meta-analysis. BMC Cancer.

[R25] Mishra SI, Scherer RW, Geigle PM, Berlanstein DR, Topaloglu O, Gotay CC, Snyder C (2012). Exercise interventions on health-related quality of life for cancer survivors. Cochrane Database Systematic Reviews.

[R26] Morey MC, Snyder DC, Sloane R, Cohen HG, Peterson B, Hartman TJ, Demark-Wahnefried W (2009). Effects of home-based diet and exercise on functional outcomes among older, overweight long-term cancer survivors: RENEW: a randomized controlled trial. Journal of the American Medical Association.

[R27] Nimptsch K, Pichon T (2015). Body fatness, related biomarkers and cancer risk: an epidemiological perspective. Hormone Molecular Biology & Clinical Investigation.

[R28] Protani M, Coory M, Martin JH (2010). Effect of obesity on survival of women with breast cancer: systematic review and meta-analysis. Breast Cancer Research & Treatment.

[R29] Rinaldi S, Peeter PH, Berrino F, Dossus L, Biessy C, Olsen A, Kaaks R (2006). IGF-I, IGFBP-3 and breast cancer risk in women. Endocrine-Related Cancer.

[R30] Schernhammer ES, Holly JM, Hunter DJ, Pollak MV, Hankinson SE (2006). Insulin=like growth factor I (IGF-I), its binding proteins (IGFBP-1 and IGFBP-3) and growth hormone and breast cancer risk in the Nurses Health Study II. Endocrine-Related Cancer.

[R31] Schmidt S, Monk JM, Robinson LE, Mourtzakis M (2015). The integrative role of leptin, oestrogen and the insulin family in obesity-associated breast cancer: potential effects of exercise. Obesity Reviews.

[R32] Shin SJ, Gong G, Lee HJ, Kang J, Bae YK, Lee A, Jung WH (2014). Positive expression of insulin-like growth factor-1 receptor is associated with a positive hormone receptor status and a favorable prognosis in breast cancer. Journal of Breast Cancer.

[R33] Short CE, James EL, Stacey F, Plotnikoff RC (2013). A qualitative synthesis of trials promoting physical activity behavior change among post-treatment breast cancer survivors. J Cancer Survivorship.

[R34] Spark LC, Reeves MM, Fieldsoe BS, Eakin EG (2013). Physical activity and/or dietary interventions in breast cancer survivors: a systematic review of the maintenance of outcomes. Journal of Cancer Survivorship.

[R35] Spector D, Deal AM, Amos KD, Yang H, Battaglini CL (2014). A pilot study of a home-based motivational exercise program for African American breast cancers: Clinical and quality-of-life outcomes. Integrative Cancer Therapy.

[R36] Stolley MR, Sharp LK, Oh A, Schiffer L (2006). A weight loss intervention for African American breast cancer survivors, 2006. Preventing Chronic Disease.

[R37] Surmacz E (2013). Leptin and adiponectin: emerging therapeutic targets in breast cancer. J Mammamary Gland Biology & Neoplasia.

[R38] Surmacz E, Otvos L (2015). Molecular targeting of obesity pathways in cancer. Hormone Molecular Biology and Clinical Investigation.

[R39] Thompson HJ, Sedlacek SM, Paul D, Wolfe P, McGinley JN, Playdon MC, Wisthoff MR (2012). Effect of dietary patterns differing in carbohydrate and fat content on blood lipid and glucose profiles based on weight-loss success of breast cancer survivors. Breast Cancer Research.

[R40] White A, Pollack LA, Smith JL, Thompson T, Underwood JM, Fairley T (2013). Racial and ethnic differences in health status and health behavior among breast cancer survivors—Behavioral Risk Factor Surveillance System. Journal of Cancer Survivorship.

[R41] Wilson DB, Porter JS, Parker G, Kilpatrick J (2005). Anthropometric changes using a walking intervention in African American BCS: A pilot study. Preventing Chronic Disease.

[R42] Vansaun MN (2013). Molecular pathways: adiponectin and leptin signaling in cancer. Clinical Cancer Research.

[R43] Yan S, Jiao X, Li K, Li W, Zou H (2015). The impact of IGF-IR expression on the outcomes of patients with breast cancer: A meta-analysis. Oncology Targeted Therapy.

[R44] Zielinska HA, Bahl A, Holly JM, Perks CM (2015). Epithelial-to-mesenchymal transition in breast cancer: a role for insulin-like growth factor I and insulin-like growth factor-binding protein 3?. Breast Cancer (Dove Med Press).

